# PKDL and Other Dermal Lesions in HIV Co-infected Patients with Leishmaniasis: Review of Clinical Presentation in Relation to Immune Responses

**DOI:** 10.1371/journal.pntd.0003258

**Published:** 2014-11-20

**Authors:** Eduard E. Zijlstra

**Affiliations:** Rotterdam Centre for Tropical Medicine, Rotterdam, The Netherlands; National Institute of Allergy and Infectious Diseases, United States of America

## Abstract

**Background:**

Co-infection of leishmaniasis and HIV is increasingly reported. The clinical presentation of leishmaniasis is determined by the host immune response to the parasite; as a consequence, this presentation will be influenced by HIV-induced immunosuppression. As leishmaniasis commonly affects the skin, increasing immunosuppression changes the clinical presentation, such as in post-kala-azar dermal leishmaniasis (PKDL) and cutaneous leishmaniasis (CL); dermal lesions are also commonly reported in visceral leishmaniasis (VL) and HIV co-infection.

**Methods:**

We reviewed the literature with regard to dermal manifestations in leishmaniasis and HIV co-infection, in three clinical syndromes, according to the primary presentation: PKDL, VL, or CL.

**Results:**

A wide variety of descriptions of dermal leishmaniasis in HIV co-infection has been reported. Lesions are commonly described as florid, symmetrical, non-ulcerating, nodular lesions with atypical distribution and numerous parasites. Pre-existing, unrelated dermal lesions may become parasitized. Parasites lose their tropism and no longer exclusively cause VL or CL. PKDL in HIV co-infected patients is more common and more severe and is not restricted to *Leishmania donovani*. In VL, dermal lesions occur in up to 18% of patients and may present as (severe) localized cutaneous leishmaniasis, disseminated cutaneous leishmaniasis (DL) or diffuse cutaneous leishmaniasis (DCL); there may be an overlap with para-kala-azar dermal leishmaniasis. In CL, dissemination in the skin may occur resembling DL or DCL; subsequent spread to the viscera may follow. Mucosal lesions are commonly found in VL or CL and HIV co-infection. Classical mucocutaneous leishmaniasis is more severe. Immune reconstitution disease (IRD) is uncommon in HIV co-infected patients with leishmaniasis on antiretroviral treatment (ART).

**Conclusion:**

With increasing immunosuppression, the clinical syndromes of CL, VL, and PKDL become more severe and may overlap. These syndromes may be best described as VL with disseminated cutaneous lesions (before, during, or after VL) and disseminated cutaneous leishmaniasis with or without visceralization.

## Introduction

**Figure 1:**
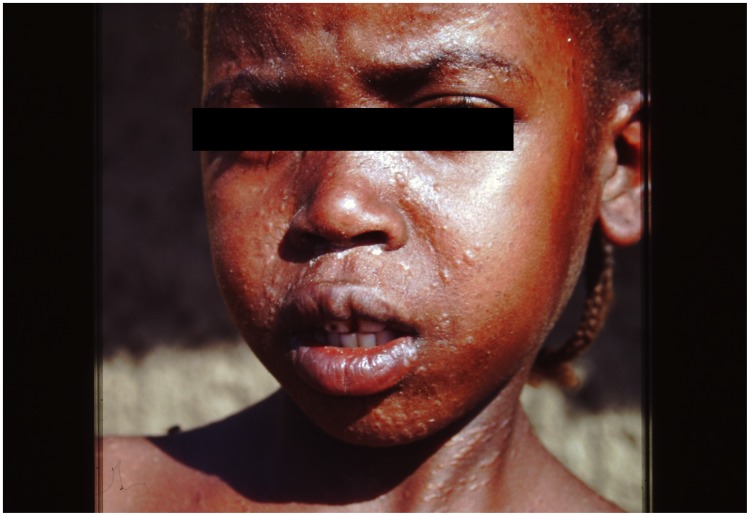
Classical PKDL from Sudan showing a papular rash on the face. The identity of the photographer was known to the patient, who gave permission. (Further images of PKDL can be found in the WHO PKDL atlas: A manual for health workers [36].)

Classically, leishmaniasis is divided into three syndromes: cutaneous leishmaniasis (CL) is the most common manifestation with 0.7–1.3 million cases annually [1]. Mucocutaneous (mucosal) leishmaniasis (MCL) is much less common; it occurs in 1%–10% of patients after a previous episode of CL [2]. Visceral leishmaniasis (kala-azar; VL) is the most serious form; annually there are 200,000–400,000 cases. Post-kala-azar dermal leishmaniasis (PKDL) may follow VL in up to 60% of cases; it is particularly important because of the presence of parasites in the skin, and patients may act as a human reservoir (Figure 1) [1], [3].

Common characteristics of all syndromes are parasite “tropism” resulting in clear delineation between clinical syndromes. This clinical presentation is the result of the interaction between the parasite and the host immune response; therefore, understanding of the underlying immunological mechanisms is important in management that may include drug therapy as well as (in combination with) immunomodulation. Involvement of the skin is the key clinical characteristic in all manifestations, except in VL, although parasites are present in the normal-looking skin. This is obviously also important in transmission.

There are many reports on leishmaniasis in immunocompromised patients; among these by far the majority of cases have been described among HIV-infected patients. Other conditions include organ transplant patients and patients with immunosuppressive or immunomodulatory therapy [4], [5]. This includes the use of topical steroids [6], [7]. Patients may become symptomatic after natural infection through the bite of a sandfly, by reactivation of a dormant infection, or by introduction of the parasite through an organ graft, in the case of transplant patients.

## HIV and Leishmaniasis Co-infection

HIV and *Leishmania* co-infection is increasingly reported; epidemiologically, the area where HIV and leishmaniasis overlapped first was in countries along the Mediterranean basin in the 1990s. Hence most reports are from VL cases with *Leishmania infantum* infection [8], [9]. Based on this experience, VL was included in the World Health Organization (WHO) Clinical Staging System for HIV, as a stage 4, AIDS-defining condition (atypical disseminated leishmaniasis) [10]. Two to nine percent of all VL cases are reported to be co-infected with HIV. Both infections mutually promote each other. The risk of VL is increased more than 2000-fold in HIV infection; conversely, VL promotes HIV replication [11], [12].

Currently most VL co-infected cases are from Ethiopia, where 40% of VL cases (*L. donovani*) are HIV positive [11]. Co-infection is increasingly reported from Brazil, where HIV spreads from urban areas to rural areas, whereas for leishmaniasis the opposite occurs, spreading from rural to urban areas; most cases affect patients with mucocutaneous leishmaniasis rather than VL [13], [14]. The parasite is *L. infantum/chagasi* in VL and *L. braziliensis* in MCL. In Asia, only sporadic cases of co-infection have been described; in India the prevalence of HIV was 5.6% among 2,077 VL patients in Bihar, where 80%–90% of all cases in India occur [15]. The parasite is *L. donovani* (India, Bangladesh) and *L. siamensis* (Thailand).

CL and HIV co-infection is reported from Africa (*L. major*), Asia (*L. tropica*), and Latin America (*L. braziliensis, L. guyanensis*).

**Figure 2:**
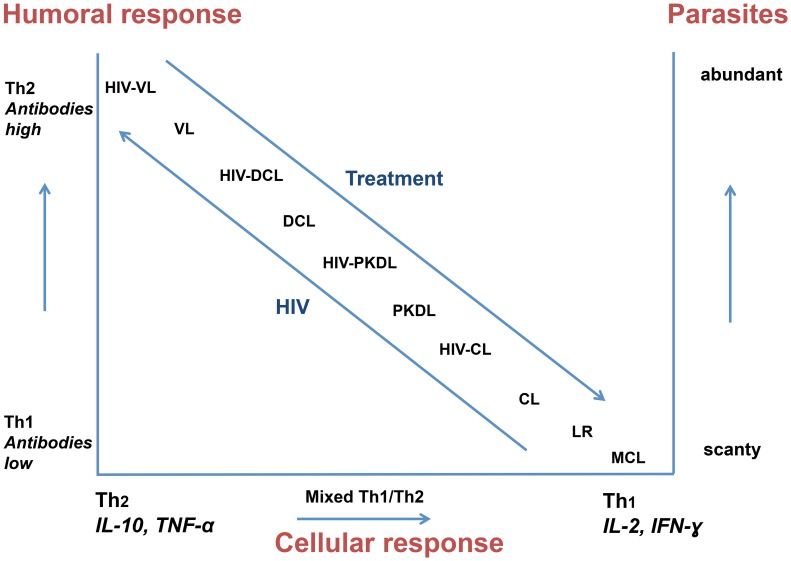
A simplified, theoretical presentation of the relationship between Th1 and Th2 responses, and parasite load, with the influence of HIV and antileishmanial and antiretroviral treatment on cutaneous leishmaniasis (CL), post-kala-azar dermal leishmaniasis (PKDL), diffuse cutaneous leishmaniasis (DCL) and visceral leishmaniasis (VL). The Th17 response is not shown but is similar in polarity to the Th1 response. Mucocutaneous leishmaniasis (MCL) and leishmaniasis recidivans (LR) are indicated as reference points.

As the clinical manifestations of leishmaniasis depend on the interaction between the (numbers of) parasites and the ensuing immune response, it follows that clinical manifestations of leishmanial syndromes that present with absent or poor cellular responses such as VL, PKDL, and diffuse cutaneous leishmaniasis (DCL) may become more common and more severe in HIV infection (Figure 2). In these conditions parasites may become more numerous, and the classical delineation between clinical syndromes becomes blurred. Parasites lose their “tropism;” for example, *L. tropica*, *L. major*, or *L. guyanensis* infections that classically cause CL have been reported to cause VL. Similarly, while skin lesions are not a feature of VL in immunocompetent patients, they are commonly described in HIV-VL and are not restricted to *L. donovani*. As a rule, the same strain that causes VL is also found in the accompanying skin lesion; however, there are a few exceptions, and new strains of *Leishmania* parasites have been isolated that do not cause disease in those who are not HIV infected [16]. In one case of para-kala-azar dermal leishmaniasis from Ethiopia, a unique Restriction Fragment Length Polymorphism (RFLP) pattern was found, while in three other patients with disseminated cutaneous leishmaniasis during VL, paired strains isolated from the skin and viscera were identical [17]. In Brazil, the strains isolated from the bone marrow and the skin lesions differed in one case [18].

## Immune Responses in CL, VL, and PKDL in Immunocompetent Patients

The immune response in leishmaniasis was clearly described in the murine model as a Th2 response in disease and a Th1 response during cure. In humans the dichotomy as found in the murine model is not so clear, and disease progression is determined by the changing cytokine profile [19]. Furthermore, a third T helper subset (Th17) was recently identified and shown to play a role in early mucosal immune defenses; so far, this subset has been implicated in cutaneous and mucosal leishmaniasis, as well as in PKDL [20]–[23].

In CL, parasites in the skin elicit an immune response characterized by a mixed Th2/Th1 response; IFN-γ, TNF-α, IL-4, IL-10, and IL-12 may all be demonstrated. The low IFN-γ levels that are down-regulated by IL-10 may play a role in the development of the lesions and parasite multiplication. Necrosis is a feature of the pro-inflammatory response. After cure, only elevated levels of IFN-γ are found; the leishmanin skin test (LST) becomes positive [24].

In contrast, in diffuse cutaneous leishmaniasis (DCL) a Th2 response is found with demonstrable IL-4, IL-5, and TNF and low levels of IL-12 and IFN-γ. The LST is negative.

In MCL, the exaggerated immune response determines the clinical presentation that is characterized by progressive tissue necrosis. It is thought that reduction of IL-10 levels or reduced expression of the IL-10 receptor contribute to overproduction of IFN-γ. Recently, it was demonstrated that IL-17 plays an important role that is produced through stimulation by IL-1β and regulated by IL-10 and IFN-γ [25]. The LST is strongly positive.

In VL, parasites can be readily demonstrated in aspirates of lymph nodes, bone marrow, or spleen. The immune response is characterized as a predominantly Th2 response with a strong humoral response with high titers of antibody; in addition, there is an absent cellular immune response to *Leishmania* parasites as evidenced in vitro by peripheral blood mononuclear cells (PBMC) stimulation and in vivo by a negative LST. The cytokine profile shows IL-4 stimulation with high levels of IL-10 and IL-13 in the peripheral blood, leading to reciprocal inhibition of TNF-α and polyclonal B-cell stimulation with high circulating antibody levels [26]. The development of a healing immune response is triggered by antileishmanial therapy and is essential for cure; macrophage activation with subsequent killing of *Leishmania* parasites plays a pivotal role. After cure of VL, a predominantly Th1 response is found that is characterized by the development of a cellular immune response to *Leishmania* parasites; stimulating PBMCs will show high levels of IL-2 and interferon-γ and the LST will become positive. The humoral immune response decreases over time with subsiding antibody levels [26]. Despite absence of parasites in aspirates of bone marrow or spleen, it is thought that there is no sterile cure in VL and that parasites persist; therefore, any subsequent immune suppression may lead to disease reactivation [26].

**Figure 3:**
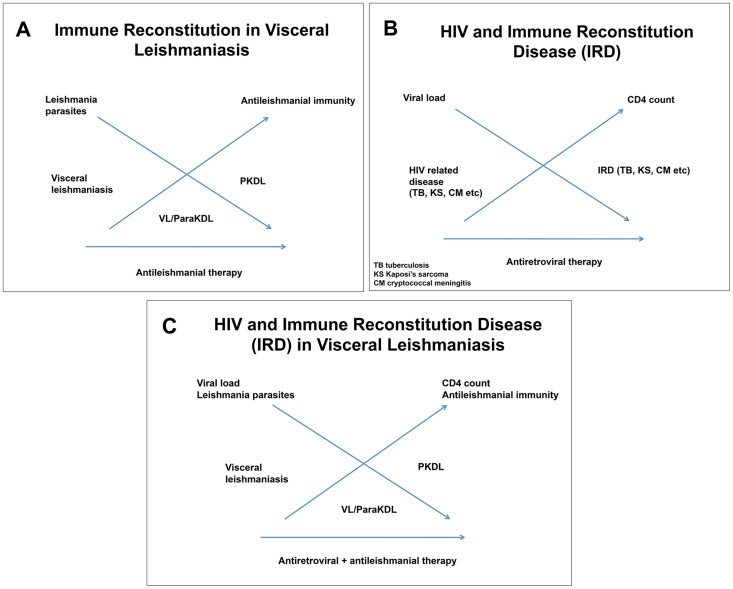
Immune (re-)constitution and post-kala-azar dermal leishmaniasis (PKDL). *Panel A*: Immune constitution in PKDL in immunocompetent patients. Theoretical relationship between changes in parasite load and immune response as the result of antileishmanial treatment, with associated clinical syndromes (visceral leishmaniasis [VL], PKDL) *Panel B*: Immune reconstitution in HIV infection. Theoretical relationship between changes in viral load and CD4 count as a result of antiretroviral therapy, with examples of associated immune reconstitution disease (cryptococcal meningitis, Kaposi's sarcoma, tuberculosis). *Panel C*: Theoretical overlap in mechanisms of PKDL occurring during VL and HIV co-infection treated with combined antileishmanial and antiretroviral therapy.

In PKDL, parasites persist in the skin after VL while they can no longer be demonstrated in the viscera; PKDL is typically the result of immune (re-)constitution (Figure 3A); in PKDL a (anti-inflammatory) mixed Th1/Th2 immune response is seen with IFN-γ production and persistence of IL-10 in the skin and in the peripheral blood. Th17 responses (through IL 17) have been demonstrated in the skin as well as in the peripheral blood [20]. In some patients, PKDL occurs while the patient is still on treatment for VL (para-kala-azar dermal leishmaniasis); PKDL can also occur in absence of previous VL, probably following subclinical infection with an adequate developing immune response preventing systemic disease. Healing of PKDL is a function of a changing immune response to a pure Th1 (pro-inflammatory) response with a pivotal role for IL-2 [26]–[28].

## Immune Responses in HIV and Leishmaniasis Co-infection

Both infections target macrophages and dendritic cells; HIV targets CD4 cells directly while *Leishmania* does this indirectly by promoting HIV replication in CD4 cells that play a pivotal role in the immune response needed to combat both infections. This leads to a predominantly and aggravated Th2 immune response. The Th17 cells subset response is lost earlier than that of Th1 cells [29]. In HIV-VL co-infection, the Th2 response that is characteristic of VL is exaggerated, with decreased levels of IL-12 and IL-18 and IFN-γ [30]. In HIV and CL co-infected patients, a similar change was not found in a study with a limited number of patients who had normal IFN-γ levels, but this change may underlie more severe cutaneous disease [31]. Patients co-infected with HIV and tegumentary leishmaniasis (the term used in South America to denote CL, MCL, or DCL) were shown to have decreased absolute numbers of effector and central memory CD4 cells with decreased function as demonstrated by response to stimulation with *Leishmania* antigen [32]; however, this defect is not as profound as in HIV-VL co-infections (>350 and <200 CD4 cells/mm^3^, for tegumentary and visceral leishmaniasis, respectively) [33]. There is an increased degree of immune activation [33]. Patients with HIV-MCL co-infection have higher levels of TNF-α than HIV-negative patients with MCL, and this may contribute to the increased severity in co-infected patients; there may be a role for modulation by Th17 helper cells [29], [34], [35].

## Dermal Lesions in Leishmaniasis Co-infected with HIV

These vary from classical localized cutaneous leishmaniasis (LCL) to more severe and atypical LCL, CL resembling disseminated cutaneous leishmaniasis (DL) or diffuse cutaneous leishmaniasis (DCL), (D)CL causing visceral disease, VL with cutaneous lesions and PKDL, or PKDL-like lesions after VL. These syndromes have been described in numerous case reports and case series; various terms have been used to describe these manifestations, while the underlying mechanism was probably similar (Table 1) [11].

**Table 1:** Examples of terminology used in the literature to describe (muco-)cutaneous involvement by *Leishmania* in HIV co-infected patients.

Terminology used
• Atypical disseminated leishmaniasis resembling PKDL
• Atypical leishmaniasis in HIV
• Cutaneous leishmaniasis associated with visceral leishmaniasis in AIDS
• Cutaneous lesions following treatment of HIV-VL
• Disseminated CL resembling PKDL in VL
• Disseminated CL after VL
• Disseminated dermal and visceral leishmaniasis in an AIDS patient
• Disseminated cutaneous leishmaniasis in AIDS
• Disseminated/visceralized CL
• Diffuse cutaneous leishmaniasis associated with IRIS
• Diffuse cutaneous leishmaniasis in HIV
• Diffusely disseminated cutaneous *L. major* infection
• Generalized cutaneous leishmaniasis in AIDS
• Kaposi's sarcoma like lesions and other nodules as cutaneous involvement in AIDS-related VL
*• Leishmania* in specific and nonspecific skin lesions in HIV-VL
• Leishmaniasis presenting as a psoriasiform eruption in AIDS
• Leishmaniasis with rheumatoid nodulosis in a patient with HIV infection
• Mucocutaneous leishmaniasis and HIV
• PKDL as immune reconstitution inflammatory syndrome
• PKDL associated with AIDS
• PKDL in HIV
• Tegumentary leishmaniasis as cause of IRIS
• Unusual association of American cutaneous leishmaniasis and acquired immunodeficiency syndrome
• Unusual cutaneous lesions in HIV-VL
• Unusual manifestations of tegumentary leishmaniasis
• VL and disseminated CL
• VL and disseminated cutaneous lesions
• VL with cutaneous lesions in HIV
• VL and para/post-kala-azar dermal leishmaniasis

In this paper we review the literature with regard to dermal manifestations of leishmaniasis in HIV–co-infected patients in three groups according to the main underlying clinical syndrome that is reported: PKDL, VL, or CL.

We searched the electronic database PubMed with the following search terms: “HIV AND post-kala-azar dermal leishmaniasis,” or “dermal leishmaniasis,” or “skin leishmaniasis,” or “visceral leishmaniasis,” or “cutaneous leishmaniasis,” or “mucosal leishmaniasis,” or “mucocutaneous leishmaniasis;” in addition: “leishmaniasis” or “leishmania AND immune reconstitution.” Case reports, case series, and reviews were all included. From the references of each article, further papers were screened.

For consistency, the following clinical definitions were used to describe the clinical presentation, whether or not influenced by HIV. Clinical images of PKDL and of dermal lesions in HIV and *Leishmania* co-infection were recently published [36].

LCL refers to the classical cutaneous leishmanial ulcer, caused by a spectrum of species but mainly caused by *L. major* and *L. tropica* in the Old World and *L. braziliensis* in the New World.

DL is distinct from LCL and DCL; it is defined by the presence of more than ten mixed-type lesions (e.g., acneiform, papular, nodular, and/or ulcerated), located in more than two body parts (head, trunk, arms, and legs). The lesions are assumed to have disseminated from a single initial lesion within 3 days to 8 weeks, and this has been described for *L. braziliensis, L. amazonensis*, and *L. guyanensis* [37], [38]. The number of lesions can be between ten and 800 [38]. The LST is positive or will become positive after treatment; response to treatment is good.

DCL is much less common than DL and classically caused by *L. amazonensis*, *L. mexicana*, and *L. pifanoi* in the Americas and *L. aethiopica* in Africa. It is characterized by specific depression of the antileishmanial immune response in an otherwise immunocompetent patient with a normal CD4/CD8 ratio, along with a high parasite load in diffuse nodular lesions, accompanied by a negative LST and a poor response to treatment [39].

Mucocutaneous leishmaniasis refers to mucosal lesions mainly affecting the nasal septum, occurring after healing of previous LCL caused by *L. braziliensis*.

Mucosal leishmaniasis refers to mucosal lesions on the oral cavity, nose, pharynx, larynx, or the eyes and may occur with LCL, VL, or PKDL; these lesions have been described for *L. donovani*, *L. infantum*, *L. major*, and *L. tropica* in the Old World and *L. braziliensis* in the New World.

PKDL refers to a skin rash that develops after treatment of VL; it is characterized by the appearance of the rash, its typical distribution, and the temporal relationship to VL (mainly in *L. donovani*).

Para-kala-azar dermal leishmaniasis is similar to PKDL, but presents during ongoing (treatment of) VL.

## Classical PKDL and other dermal lesions following HIV co-infection

There is little overlap between areas with significant PKDL rates and HIV; in Sudan where PKDL is most common, HIV prevalence is 1.3%; VL mainly affects children [40]. Few cases of co-infection of HIV and VL or PKDL have been described in the main endemic area in eastern Sudan. This endemic focus continues across the Ethiopian border as the Metema-Humera endemic area; here VL is commonly seen among young male agricultural workers who originate from the Ethiopian Highlands and in whom a HIV prevalence rate of 40% has been reported [41]. In India, only one of 24 PKDL cases was HIV infected during a 5-year follow-up study of VL patients [42]. Sporadic cases have been reported from the Americas, Europe, Asia, and the Middle East [18], [43]–[55].

One study found PKDL to be more common (and more severe) in those who were HIV positive as compared to HIV-negative patients; 27% versus 13% [41]. Clinical manifestations in co-infected patients with PKDL may differ (Table 2). Most patients have florid disease with various descriptions including macules, (painful) papulo-erythematous eruption, disseminated miliary papules, nodules and plaques, or a mixed picture; in most, parasites were easily demonstrated [43]–[47], [49], [53]–[56]. The classical distribution and spread from the face to other parts of the body is not always found. Lesions are often reported on the acra as well as other parts of the body (Table 2), often with abundant parasites [57]. Ulceration is not a feature, but scrotal ulcers have been reported. There may be concomitant uveitis [43], [46]. In one report, the only manifestation was an elevation of a tattoo from which parasites could be isolated [50]. The clinical picture may not resemble PKDL despite the occurrence of lesions after VL treatment. In one report, a patient developed DCL 13 months after VL treatment. In another case from Brazil, a papular rash developed after multiple VL episodes with another subsequent VL episode; intercurrent CL was considered as a possibility, as typing was not done [58], [59]. PKDL in co-infected patients is not restricted to *L. donovani* because cases of *L. infantum/L. chagasi* have been reported from the Mediterranean area as well as from Latin America [43], [44], [47], [48], [52]. In some case reports, patients have had multiple courses of treatment for VL, and PKDL occurs in the context of IRD (see below), although this could have been ascribed to antileishmanial therapy only. In others, the HIV infection was detected only at the time of presentation with PKDL [51]. Skin lesions have also been described as the first manifestation of a concomitant recurrence of VL; hence, the ensuing clinical syndrome may be referred to as para-kala-azar dermal leishmaniasis [60]. All patients reported with PKDL and HIV co-infection had a CD4 count of less than 350 mm^3^; in 95% this count was less than 200 mm^3^.

**Table 2:** Comparison of PKDL and PKDL-like lesions in immunocompetent and in immunocompromised patients.

	Immunocompetent	Immunocompromised
**Parasite**	*L. donovani*, mainly	Also *L. chagasi*/*L. infantum*
**Frequency**	less frequent, less severe than in immunocompromised	more frequent, more severe than in immunocompetent
**Main clinical presentation**	macular or maculopapular	nodular
**Other post-kala-azar manifestations**	yes, uveitis	yes, uveitis
**Post- or para-KDL**	post>> para	para>> post
**Parasites numbers**	scanty	abundant
**Parasites found in skin**	<60%	90%
**Ulcerating**	no	no, but genital ulcers described
**Face affected**	almost always	not always
**Acra involved**	no	often; symmetrical
**Evolution**	typical	atypical

## PKDL-like skin and mucosal manifestations in HIV-infected patients primarily presenting with VL

While the clinical presentation of VL is largely similar in immunocompetent and immunocompromised patients, atypical presentations occur. This makes clinical suspicion difficult as the clinical presentation of advanced HIV/AIDS and VL (including the effect of medication used) may overlap (splenomegaly, lymphadenopathy, pancytopenia, etc.). In contrast to immunocompetent patients, skin manifestations are not uncommon and may precede or predict recurrence [59], [61]. In HIV-VL patients, skin lesions confirmed as caused by leishmaniasis were reported in 9%–18% and oral lesions in 3% [62], [63].

In HIV-VL co-infection with disseminated, diffuse, or generalized cutaneous leishmaniasis, the clinical syndrome is similar to DCL but the CD4 count is low (usually <200/mm^3^) and the LST is usually negative [17], [60], [64], [65]. One report of DCL in HIV co-infection mentioned a positive LST and good response to treatment [66]. Parasites may usually be demonstrated without much difficulty and may be other species than those classically associated with DCL [60], [67]. This syndrome has been described for a number of leishmanial parasites, including *L. siamensis* [68].

Basically, these cases are similar to para-kala-azar dermal leishmaniasis, but lesions are predominantly nodular and more florid and more extensive; some have atypical clinical presentations and a distribution similar to what has been described for PKDL in HIV co-infection (Table 2). As in PKDL, ulceration is not a feature, but pruritus has been described [62]. Subcutaneous nodules may also be found [62], [68], [69]. Acral distribution is common; hyperpigmentation and scaling may occur [16], [62]. It may be difficult to distinguish between PKDL and para-kala-azar dermal leishmaniasis when skin lesions occur after multiple episodes of treatment of VL but with evidence of visceral disease still remaining [70]. Other even more atypical lesions have been described, such as linear macules on the palms, an erythrodermic pattern, and lesions resembling psoriasis and erythematoviolaceous plaques [50], [62], [71], [72]. Some lesions resemble Kaposi's sarcoma [57]. Concomitant gastrointestinal lesions may be found. Mucosal lesions may occur, e.g., on the tongue, oral mucosa, oropharynx, larynx, and penis [50], [73], [74].

Skin lesions do not only occur de novo but have been described in pre-existing conditions such as Kaposi's sarcoma, dermatofibroma, herpetic lesions (including herpes zoster), tattoos, cryptococcosis, atypical mycobacteria lesions, bacillary angiomatosis, and oral aphthae [14], [50], [60], [71], [75]–[78]. In the case of dermatofibroma, it has been suggested that this may be a new manifestation of dermal kala-azar with multiple dermatofibromas developing; alternatively, it may be a purely co-incidental finding or it may reflect the sequestration of leishmanial parasites through an existing dermatofibroma, thus producing favorable conditions for parasite survival [77], [79]. A similar mechanism has been suggested by demonstration of the presence of leishmanial parasites in secretory eccrine glands in normal skin, in a dermatomyositis-like presentation, and in cutaneous leishmaniasis caused by *L. major* and *L. tropica* [65], [80]–[82]. This supports the suggestion that HIV-VL co-infected patients, because of the high parasite load with circulating parasites in the peripheral blood and abundant parasites in the skin, may play an important role in transmission. It should be noted that parasites may be also be found in completely healthy-looking skin [57], [65], [71], [80], [81], [83], [84].

## (PKDL-like) dermal manifestations in HIV-infected patients primarily presenting with skin lesions suggesting cutaneous leishmaniasis or mucosal leishmaniasis or who are from a non-VL endemic area

The clinical manifestations of cutaneous leishmaniasis and mucocutaneous leishmaniasis may differ in HIV infection with the degree of reduction in CD4 count as an important factor. For CL, while in some reports no difference in clinical manifestations is seen between immunocompetent and immunosuppressed patients, in others, more lesions or more severe lesions are seen [85]–[87]. In a study from French Guyana, HIV-infected patients had similar localized CL as in matched HIV-negative controls with 1 MCL case (all had *L. guyanensis* infection; all HIV infected patients had a CD4 count of > 200 mm^3^). They differed in higher recurrence rates and more difficulty to treat [88]. Similar clinical findings were found in a later study with CD4 counts in the range of 35–612 cells/mm^3^ [89]. Cutaneous leishmaniasis mimicking dermatomyositis has been described [81]. In a study from Burkina Faso in 32 patients with CL supposedly caused by *L. major*, patients presented with multiple lesions and a polymorphic picture that resembled other conditions such as psoriasis, leprosy or Kaposi's sarcoma [90]. There seems to be no uniform clinical presentation, but symmetrical acral lesions are commonly seen. Macules, papules, nodules, and plaques or combinations have been described. Ulceration seems less prominent than in immunocompetent patients; sometimes a mixed picture is seen [6].

**Figure 4:**
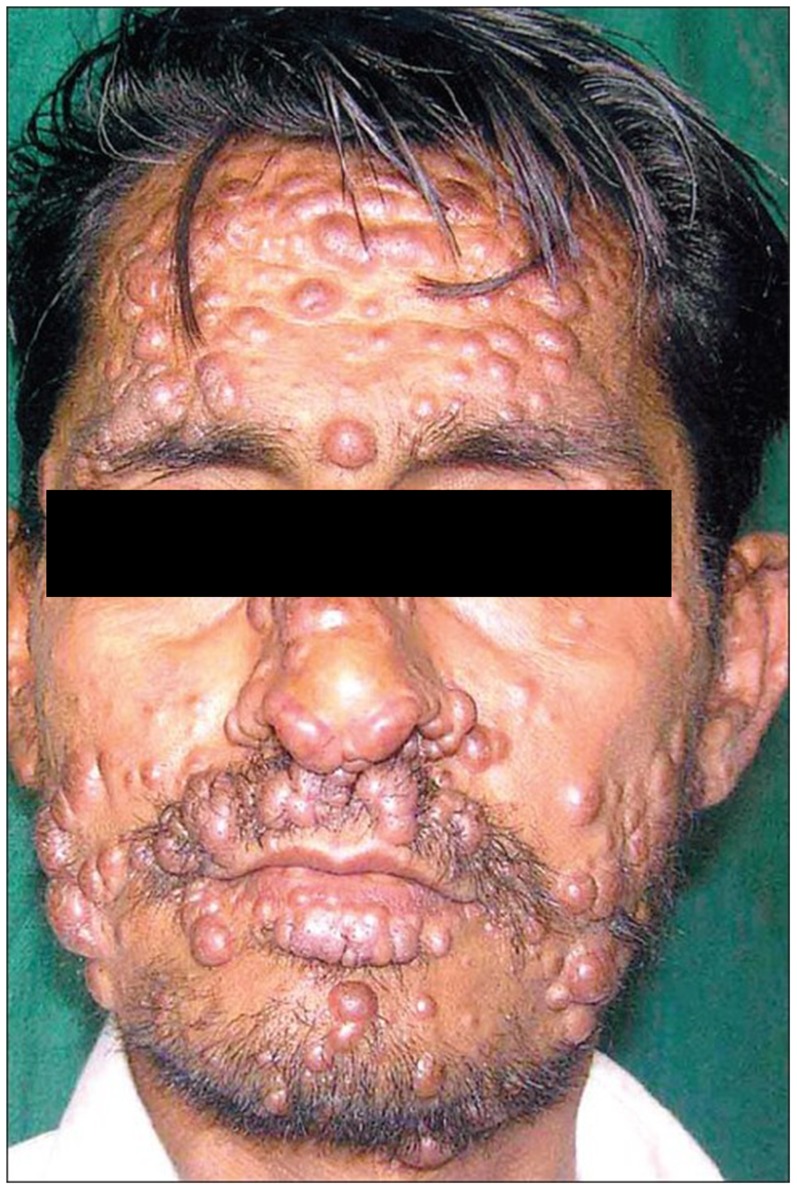
Diffuse or disseminated cutaneous leishmaniasis in an HIV-positive patient from Rajasthan, India (endemic for CL, not VL). There are florid nodules mainly on the face and on the extremities and back; oral and nasopharyngeal infiltrations were also found. There was no previous ulcer nor were there signs of visceralization. The parasite was not typed. Reproduced with permission from Chaudhary et al., the Indian Journal for Dermatology, Venereology and Leprology [97].

With decreasing CD4 counts, unusual manifestations occur. In a study from Brazil (*n*  =  15, mean CD4 count 84/mm^3^) the spectrum ranged from single to multiple and polymorphic lesions, with mucosal lesions in 80%, cutaneous lesions in 73% (53% with mucocutaneous lesions), disseminated lesions in 60%, and genital ulcers in 27%; bone marrow aspirates were negative [91]. Ulceration becomes less prominent and multiple (confluent) nodules and plaques are more common [92]. This may lead to a clinical picture of disseminated cutaneous leishmaniasis (DL) or diffuse cutaneous leishmaniasis (DCL) (Figure 4) [93]. While in immunocompetent patients DL may be characterized by ulceration and a positive LST, in HIV co-infection the clinical picture changes, with numerous (10–300) pleiomorphic lesions and resemblance to DCL [94]. The lesions are papulonodular, non-ulcerating, contain abundant parasites, and are not painful but may be intensely pruritic. Some of the criteria for DCL may be found (immunocompetent, specific anergia against *Leishmania*, negative LST, no response to treatment) although these patients are immunosuppressed, and in some, a positive LST was found [66], [95]. In others, the diagnosis of DCL caused by primary CL aggravated by HIV co-infection was supported by the fact that the patient was from a CL-endemic area [96]–[99]. This dissemination in the skin has been described for virtually all *Leishmania* species [85], [100], [101]. In endemic areas, DCL is often confused with lepromatous leprosy, and it may be the first indicator of HIV infection [98]. DCL may be restricted to the skin or only cause mucosal spread despite the potential of spread, such as in the case of *L. tropica* or *L. infantum* [91], [98], [102].

In others, visceralization occurs that can be demonstrated by finding leishmanial parasites (or leishmanial DNA by PCR) in bone marrow or spleen aspirates; in addition, several cases of spread to the gastrointestinal mucosa have been described and parasites may be found in the peripheral blood. This has been mainly described for *L. infantum/chagasi* but may also occur in disseminated cutaneous leishmaniasis (presumably) caused by *L. major*, *L. tropica*, and *L. siamensis* [6], [81], [103]–[106]. In one HIV-positive case on ART from Thailand, dissemination of a papular rash was caused by prolonged use of topical steroids [6].

In many case reports, no confirmation of visceralization was obtained despite the clinical suspicion of systemic disease and/or the presence of hepatosplenomegaly. The distinction between primary CL with subsequent visceralization and primary visceralized disease with skin lesions is blurred, even if the patient is from a CL-endemic area. Evidence of visceralization seems a function of CD4 count, duration of the disease, and rigorous examination. Lesions may be found in pre-existing conditions as described above (under the previous subheading). Parasites involved include *L. major*, *L. tropica*, and *L. infantum/chagasi* (Table 3).

**Table 3:** Clinical entities particular to HIV and *Leishmania* co-infection and with reported causative parasite and region: a new terminology based on the presence and time of presentation of VL in relation to the development of the cutaneous lesions.

Disseminated cutaneous leishmaniasis without VL (history or present):
* • L. major* (Burkina Faso, Ghana)
* • L. tropica* (India)
* • L. chagasi* (Nicaragua)
* • L. guyanensis* (French Guyana)
* • L. infantum* (France)
Disseminated cutaneous lesions preceding VL (*pre-kala-azar dermal leishmaniasis*)*
* • L. infantum* (France)
Disseminated cutaneous lesions with concomitant VL (*para-kala-azar dermal leishmaniasis*)
* • L. donovani* (Ethiopia, India)
* • L. infantum* and *L. donovani* (Brazil)
* • L. infantum* (France)
* • L. siamensis* (Thailand)
* • L. tropica* (Iran)
* • L. major* (Burkina Faso)
Disseminated cutaneous lesions after VL (*post-kala-azar dermal leishmaniasis*)
* • L. donovani* (Ethiopia, India)
* • L. infantum/L. chagasi* (France, Italy, Greece, Spain, Portugal, Brazil, Honduras)
* • L. major* (Burkina Faso)

The corresponding terminology with PKDL as a starting point is indicated.

*can only be diagnosed retrospectively

Mucosal lesions are not uncommon in co-infected patients, either with primarily CL or VL. Lesions may appear on the tongue, buccal mucosa, oropharynx, and larynx [87], [107]–[112]. Combined CL and mucosal involvement has been described in HIV co-infected patients with *L. braziliensis*, *L. infantum*, or *L. major* infection and *L. guyanensis*; the absence of a positive LST should alert one to the possibility of HIV co-infection [38], [113]–[115].

Classical MCL caused by *L. braziliensis* infection is more severe in co-infection with more extensive lesions, such as diffuse infiltration of the palate; concomitant genital ulceration is common (27%) [35], [91], [114], [116]. MCL has also been described in (not-imported) patients in Europe, and in some, the parasite was typed as *L. infantum* [117]–[120].

## Immune Reconstitution Disease

In HIV-positive patients, the immune reconstitution disease (IRD) or the immune reconstitution inflammatory syndrome (IRIS) follows immune reconstitution as the result of antiretroviral therapy in HIV-infected patients (Figure 3). It occurs in 10%–32% of patients on ART [121]. Certain criteria should be fulfilled: (1) diagnosis of AIDS; (2) on ART with increase of CD4 and decrease in viral load; (3) development of inflammatory disease while on ART; and (4) features cannot be ascribed to side effects of therapy, new infection, or previously recognized infection [121]. Typically, the abrupt shift from an anti-inflammatory state with low CD4 counts to a pro-inflammatory state with increased CD4 count, reduced T-regulatory cells, and exaggerated cytokine responses may result in clinical manifestations of various infections; most commonly mycobacteria, fungi, viruses (herpes simplex virus, hepatitis viruses, cytomegalovirus), *Pneumocystis jerovici* Pneumonia (PcP), and Kaposi's sarcoma.

In HIV and leishmaniasis co-infections, IRD is uncommon [122]. The interval between start of ART and IRD in leishmaniasis varies between 2 weeks and 4–8 months [44].

In leishmaniasis, particularly in PKDL, IRD may be difficult to appreciate as PKDL in itself is immune (re-)constitution disease (Figure 3). It is therefore conceivable that antileishmanial therapy and antiretroviral therapy both contribute to this phenomenon (Figure 3). PKDL has been reported to occur as a likely IRD in various cases reports [43]–[47], [53], [54], [56], [123]; in some, this was documented in the context of an increasing CD4 cell count as well as decreasing IL-10 and production of interferon-γ [45], [123]. Sometimes the relationship was not so clear and the lesions occurred after years on ART [46], [47]; in others, VL occurred while on successful ART and PKDL occurred after antileishmanial therapy, while ART continued [48], [49]. It may be argued that in some cases with undetectable viral load but persistent low CD4 counts, treatment of concurrent *Leishmania* infection, rather than the ART, may induce immune reconstitution resulting in PKDL [48].

IRD in relation to VL has been reported; two cases with asymptomatic infection progressed to symptomatic visceral leishmaniasis [124]–[128].

IRD has also been reported in CL (uveitis by *L. major*) and disseminated CL, with mucosal lesions starting de novo during IRD in one patient from Brazil (parasite not typed) and with worsening of pre-existing lesions in a second patient (*L. braziliensis*) [129], [130]. Lastly, IRD has been described presenting with DCL in the United States (*L. chagasi*, originally from Nicaragua and Guatemala) [131]. In another patient from Brazil, the clinical picture at presentation was that of DCL caused by *L. guyanensis* and the patient was successfully treated; later, classical LCL occurred as CD4 cells increased as a result of ART [132]. From Senegal, a patient was reported who presented with a CL ulcer with sporotrichoid distribution (*L. major*), who developed subcutaneous nodules after ART [133].

Conjunctivitis and uveitis have also been described following VL and treatment with antileishmanial and antiretroviral therapy [54], [129]. In one case, there was concomitant PKDL and anterior uveitis, and in another case, PKDL and posterior uveitis [43], [46].

## Discussion

The clinical picture of cutaneous involvement or PKDL-like lesions in HIV co-infection is predominantly characterized by multiple, florid, non-ulcerating, mainly nodular lesions with abundant parasites, often with atypical distribution and evolution. The absence or presence of ulceration may reflect the weakened immune response against the parasites. The primary underlying clinical syndrome may be that of CL, VL, or PKDL, but there is considerable overlap as the classical presentations of these conditions become increasingly unclear with increasing immunosuppression.

The diverse clinical descriptions in the literature of leishmaniasis and HIV co-infection can be summarized in four groups or clinical entities; this is shown in Table 3 with the causative parasites that have been reported in the literature. Thus, a more consistent terminology is presented that is determined by the presence or absence and timing of visceral disease in relation to the development of cutaneous lesions. It should be noted that typing and comparison of parasites from viscera and skin should ideally be performed to exclude a novel or concomitant, second leishmanial infection.

While multiple skin lesions may be the result of multiple sandfly bites or lymphatic spread with subsequent visceralization, the opposite is also possible, with primary visceral disease and dissemination to the skin through the lymphatics or hematogenous [58]. This issue of CL lesions visceralizing to VL or a primary VL case presenting with skin lesions is further complicated by the fact that most HIV-VL co-infected patients described in the literature are from Spain and possibly became infected by sharing needles in intravenous drug use, whereas all other patients probably became infected in the conventional way by the bite of a sandfly [11], [91]. In the latter, it is not clear whether multiple lesions are the result of separate sandfly bites or dissemination from a primary lesion (local, lymphatic, hematogenous).

In VL, spread occurs to the skin through the lymphatics or blood; the symmetry of lesions does not support multiple primary skin lesions but indicates an immunologically mediated mechanism. It is not clear to what extent the Köbner phenomenon may play a role. In others, the only dermal manifestation is in a pre-existing lesion such as a tattoo or a dermatofibroma; these lesions may persistently attract macrophages, including those that are infected with *Leishmania*.

In those on treatment for VL-HIV co-infection, PKDL lesions may occur; as PKDL is in itself an immune (re-)constitution phenomenon, it is not always clear whether the PKDL lesions are caused by antileishmanial therapy, antiretroviral therapy leading to IRD, or both. For the diagnosis of IRD, sequential CD4 counts and viral load measurements are necessary.

## Conclusion

The diagnosis of dermal or PKDL-like lesions in HIV co-infected patients is important because skin lesions contain abundant parasites that may be infectious to sandflies and thus may contribute to transmission. This diagnosis poses additional challenges for treatment as the penetration of antileishmanial drugs in the skin is largely unstudied, and parasites may persist despite seemingly successful treatment of VL. The poor immune responses in co-infected patients as evidenced by persisting low CD4 counts may influence this. Similarly, the skin may therefore be a source of relapse. It remains to be seen whether drug treatment of VL eradicates parasites in the skin equally to those in the viscera or if the immunological environment in the skin is different, with a subsequent different outcome to treatment.

In addition to a high parasite load and circulating parasites in the peripheral blood, abundance of parasites in concomitant dermal lesions with evidence of active excretion of parasites, such as in dermatofibromas, suggests that HIV co-infected patients with leishmaniasis may play an important role in transmission; early recognition and treatment with antileishmanial and antiretroviral therapy is therefore essential for control.

**Top Five Papers**

1. Antinori S, Longhi E, Bestetti G, Piolini R, Acquaviva V, et al. (2007) Post-kala-azar dermal leishmaniasis as an immune reconstitution inflammatory syndrome in a patient with acquired immune deficiency syndrome. Br J Dermatol 157:1032-1036. Epub 2007/09/15. (*includes a review of other published cases*)
2. Puig L, Pradinaud R (2003) *Leishmania* and HIV co-infection: dermatological manifestations. Ann Trop Med Parasitol 97 Suppl 1:107-114. Epub 2003/12/18.
3. Khalil EA, Khidir SA, Musa AM, Musa BY, Elfaki ME, et al. (2013) Post-Kala-Azar Dermal Leishmaniasis: A Paradigm of Paradoxical Immune Reconstitution Syndrome in Non-HIV/AIDS Patients. J Trop Med:275253. Epub 2013/05/02.
4. Wolday D, Berhe N, Britton S, Akuffo H (2000) HIV-1 alters T helper cytokines, interleukin-12 and interleukin-18 responses to the protozoan parasite *Leishmania donovani*. AIDS 14:921-9. Epub 2000/06/15.
5. Zijlstra EE, Musa AM, Khalil EA, el-Hassan IM, el-Hassan AM (2003) Post-kala-azar dermal leishmaniasis. Lancet Infect Dis 3:87-98. Epub 2003/02/01.
